# Cell-type-dependent decline of IGF1 and IGF1R in the prefrontal cortex contributes to age-related behavioral changes

**DOI:** 10.3389/fnagi.2026.1858227

**Published:** 2026-08-19

**Authors:** Shuwen Yue, Christian Young, Arushi Garg, Tyler Sarovich, Lu Chen, Kendall Dickerson, Zi-Jun Wang

**Affiliations:** 1 Department of Pharmacology and Toxicology, School of Pharmacy, University of Kansas, Lawrence, KS, United States; 2 Cofrin Logan Center for Addiction Research and Treatment, University of Kansas, Lawrence, KS, United States

**Keywords:** aging, anxiety-like behaviors, cell-type specificity, cognitive function, insulin-like growth factor 1, prefrontal cortex, sociability

## Abstract

Insulin-like growth factor 1 (IGF1) signaling plays a critical role in brain function and declines significantly with aging. However, how these changes occur across different brain regions and cell types, and how they contribute to behavioral dysfunction, remains poorly understood. The prefrontal cortex (PFC), a key regulator of cognitive, emotional, and social behaviors, is particularly vulnerable to age-related changes. Here, we investigated age-associated changes in IGF1 and its receptor (IGF1R) in major neuronal populations of the PFC and examined their functional relevance. Using immunohistochemical analysis across young adult, middle-aged, and aged mice, we identified cell-type-dependent alterations in IGF1 and IGF1R expressions in the PFC of C57BL/6 mice, characterized by a reduction of IGF1 in both excitatory (CaMKII^+^) and inhibitory (GAD67^+^) neurons, and a selective decrease of IGF1R in excitatory neurons. To determine the functional consequences of these changes, we employed an excitatory neuron-specific *Igf1r* conditional knockdown (cKD) model in the PFC of young adult mice. IGF1R cKD in excitatory neurons of young adult mice resulted in behavioral dysfunction across multiple domains, including increased anxiety-like behavior, reduced sociability, and impaired cognitive performance. Furthermore, chronic intra-PFC infusion of IGF1 alleviated age-associated behavioral impairments in aged mice, and these recovery effects depend on IGF1R in excitatory neurons. Together, these findings support a critical role for region- and cell type-specific IGF1 and IGF1R system in regulating behavioral function during aging and provide insight into mechanisms underlying age-associated behavioral declines.

## Introduction

Insulin-like growth factor 1 (IGF1) and its receptor IGF1R are widely expressed across multiple tissues, and their signaling declines substantially across the lifespan. In the periphery, predominantly liver-derived circulating IGF1 decreases progressively with age in both rodents and humans, largely driven by reduced growth hormone (GH) signaling (Sonntag et al., 1999; Toth et al., 2022; Wennberg et al., 2018; Sonntag et al., 2005). Consistent with systemic changes, IGF1 and IGF1R expressions are also altered in the aging brain. Animal studies have demonstrated reduced IGF1 at the whole-brain level with aging, accompanied by a corresponding upregulation of IGF1R expression, possibly as a compensatory response (Muller et al., 2012). Beyond normal aging, disrupted brain IGF1 and IGF1R levels have also been implicated in the pathogenesis of many age-related neurodegenerative diseases, including Alzheimer’s disease (AD). In the central nervous system (CNS), decreased IGF1 and IGF1R expressions have been reported in several brain regions of individuals with AD, including cortex, hippocampus and hypothalamus (Rivera et al., 2005; Steen et al., 2005). This central deficiency may result from dampened local production, as indicated by decreased IGF1/IGF1R mRNA expression (Westwood et al., 2014), or impaired peripheral input, as indicated by a lower cerebrospinal fluid/plasma IGF1 ratio (Trueba-Saiz et al., 2013). Mechanistically, diminished IGF1 signaling has been proposed to contribute to AD pathology in part by impairing the clearance of amyloid-*β* (Aβ) (Carro et al., 2002), a key factor underlying cognitive decline (Hardy and Selkoe, 2002). However, despite these observations, it remains unclear how aging alters IGF1 and IGF1R expressions within specific brain regions and cell types, and whether such changes contribute to behavioral decline during normal aging.

The beneficial effects of IGF1 on brain function have been demonstrated in many preclinical studies. Higher central level of IGF1 has been implicated in sustaining synaptic plasticity in aged mice (Sonntag et al., 2000; Maher et al., 2006). IGF1 levels are increased locally in the hippocampus of Ames dwarf mice (Sun et al., 2005a; Sun et al., 2005b), a long-lived mouse model characterized by preserved cognitive function during aging (Brown-Borg et al., 1996; Bartke and Brown-Borg, 2004). Despite markedly reduced circulating IGF1 due to diminished GH signaling, the abundance of brain IGF1 in these mice is thought to result from enhanced local production. Other studies directly manipulated IGF1 signaling to determine its causal role in regulating brain function. For example, central IGF1 treatment in aged mice improves cognitive function, as demonstrated by enhanced performance in behavioral tests assessing spatial and recognition memory (Markowska et al., 1998). IGF1 also exerts antidepressant-like and anxiolytic-like effects, evidenced by reduced immobility in the forced swim test and increased center time in the open field (Farias Quipildor et al., 2019), indicating its protective effects extend beyond cognition to affective behavioral processes during aging. Apart from exogenous IGF1 administration, gene manipulation by brain-specific IGF1 overexpression also yields a similar beneficial effect on cognitive function (Farias Quipildor et al., 2019).

However, the role of IGF1/IGF1R in aging and neurodegeneration is not clear-cut. Declining circulating IGF1 is associated with extended lifespan in rats (Mao et al., 2018), possibly due to enhanced resistance to oxidative stress and increased cellular resilience (Murakami, 2006). Reduced IGF1R signaling has also been reported to provide neuroprotection in AD by attenuating neuroinflammation and neuronal loss in AD model mice (Cohen et al., 2009). This complexity may be partly attributed to bulk tissue analyses and manipulation that overlook cell-type and regional heterogeneity. Therefore, studies using cell-type-specific and spatially resolved methods are needed to clarify the role of IGF1 and IGF1R in aging.

The prefrontal cortex (PFC) is particularly vulnerable to age-related changes, and its dysfunction is closely tied to the hallmark behavioral deficits of aging, including impairments in cognitive, emotional, and social behaviors (West, 1996; Prakash et al., 2009; Bloss et al., 2010). Downregulation of NMDA receptor NR2B expression in the PFC with advanced age may contribute to cognitive impairment by reducing persistent network activity (Wang et al., 2013; Wang et al., 2011), a key neural mechanism underlying working memory performance (Constantinidis et al., 2018). Consistent with these circuit-level changes, aging is associated with a selective loss of thin dendritic spines in the PFC, presumably the most plastic spines in response to learning (Bourne and Harris, 2007). These synaptic alterations correlate with declines in memory performance, suggesting that loss of synaptic plasticity contributes to age-related PFC dysfunction (Dumitriu et al., 2010). Beyond cognition, age-associated increases in anxiety-like behavior have been linked to structural remodeling of medial PFC circuits, including dendritic and morphological changes (Sotoudeh et al., 2020). Similarly, age-related declines in social behavior have been associated with reduced excitatory neuron activity in the aging PFC, which contributes to dampened social dominance in aged mice (Shan et al., 2023). Taken together, these findings suggest that age-related alterations in synaptic and circuit function in the PFC may underlie behavioral changes across multiple domains. In line with this, our previous work demonstrated that IGF1 regulates excitatory synaptic transmission in PFC pyramidal neurons (Yue et al., 2022), raising the possibility that age-related altertions in IGF1 signaling in the PFC may contribute to behavioral changes, which remains to be determined.

To address these questions, we first tested age-associated changes in IGF1 and IGF1R expressions in excitatory and inhibitory neurons in the prelimbic (PrL) region of the PFC in young adult, middle-aged, and aged mice. Using immunohistochemistry, we identified a divergent alteration in IGF1 and IGF1R expressions, characterized by a reduction of IGF1 in both calcium/calmodulin-dependent protein kinase II-positive (CaMKII^+^) and glutamate decarboxylase 67-positive (GAD67^+^) neurons and a selective decrease of IGF1R in CaMKII^+^ neurons. We next examined whether this age-associated disruption of IGF1 and IGF1R expressions in the PrL contributes to behavioral alterations by selectively knocking down IGF1R expression in PrL excitatory neurons in young adult mice. This manipulation resulted in increased anxiety-like behaviors, reduced sociability, and impaired cognitive function. Finally, we tested whether local IGF1 infusion in the PrL of aged mice could ameliorate age-associated behavioral alterations and whether its action depends on IGF1R in excitatory neurons. Together, these findings reveal that IGF1 and IGF1R in the PrL are regulated in a cell-type-dependent manner during aging and suggest that their disruption in excitatory neurons contributes to age-associated behavioral alterations.

## Methods

### Animal

C57BL/6 J mice (#000664) and *Igf1r*^flox/flox^ (*Igf1r*^f/f^) mice (B6;129-*Igf1r^tm2Arge^*/J, #012251) were purchased from Jackson lab. Both male and female mice were used in this study. All animal experiments were performed under the approval of the Institutional Animal Care and Use Committee, University of Kansas. All animals were maintained according to the National Institutes of Health guidelines in Association for Assessment and Accreditation of Laboratory Animal Care accredited facilities.

### Immunohistochemistry and quantifications

Three age groups were used: young-adult (2 months), middle-aged (9 months) and aged mice (15–18 months). Mice were transcardially perfused, and brains were collected and sectioned for IGF1 and IGF1R staining. IGF1 and IGF1R immunofluorescence intensities in the CaMKII^+^ and GAD67^+^ neurons within the PrL were quantified in ImageJ by identifying cells positive for the respective markers, as previously reported (Yue et al., 2022; Wang et al., 2025). Immunofluorescence signals were averaged across four images per animal to obtain a single value for each animal (*n* = 4 animals/group), which was used for statistical analysis. Detailed procedures are described in the Supplementary materials.

### Stereotaxis surgery and IGF1 microinjection

To induce *Igf1r* conditional knockdown (cKD), rAAV-CaMKIIa-mCherry-P2A-CRE-WPRE-hGH polyA (titer: 2×10^12^ VG/mL, #PT-0739, BrainVTA) or control virus rAAV-CaMKIIa-mCherry-WPRE-hGH polyA (titer: 2×10^12^ VG/mL, #PT-0108, BrainVTA) was delivered bilaterally (300 nL/side) into the PrL (AP: +2.0, ML: ±0.3, DV: −2.0) of *Igf1r^f/f^* mice. Behavioral experiments or tissue collection were performed after 14 days of viral expression. For IGF1 infusion, cannulation was performed at the same time as viral injection. Mice were assigned to receive saline (VEH) or IGF1 (100 ng/μL) infusion (1 μL/hemisphere, 0.5 μL/min). Detailed surgical procedures are described in the Supplementary materials.

### Behavioral test

Animals were subjected to a series of behavioral tests in the order shown in Supplementary Figure 2A, including open field, light–dark box, elevated plus maze, social interaction, three-chamber social preference, and novel object recognition. Detailed behavioral test procedures are described in the Supplementary materials.

### Statistical analysis

All statistical analyses were performed with GraphPad Prism 9.0 and Statistical Package for the Social Sciences (SPSS). Data were analyzed using one-way or two-way ANOVA as appropriate, followed by Bonferroni *post hoc* tests for multiple comparisons. Experiments with two groups were analyzed using two-tailed unpaired *t*-tests. All data are presented as the mean ± SEM, with *p* < 0.05 indicating significance. The number of animals in each group was provided in figure legends. Statistical details are provided in Supplementary Table 1.

## Results

### Aging induces cell type-dependent reductions of IGF1 and IGF1R expressions in the PrL

**Figure 1:**
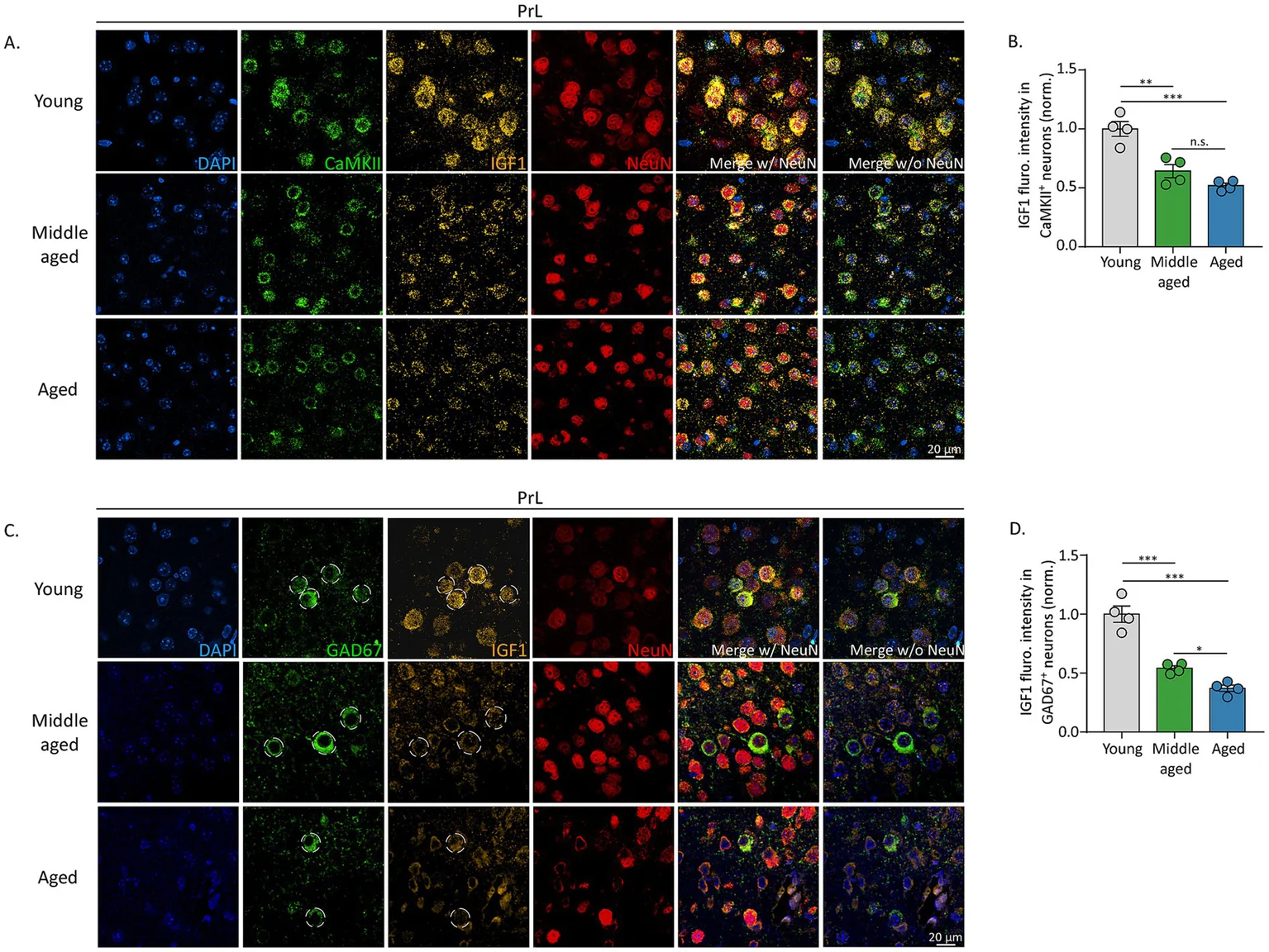
Aging decreases IGF1 expression in GAD67^+^ and CaMKII^+^ neurons in the PrL. **(A)** Representative immunohistochemical images showing IGF1 protein level in CaMKII^+^ and NeuN^+^ neurons from the brain sections containing the PrL. **(B)** Quantification of IGF1 immunofluorescence intensity in CaMKII^+^ neurons (*n* = 4 mice/group, one-way ANOVA, *F*_(2, 9)_ = 25.18, *p* = 0.0002). **(C)** Representative immunohistochemical images showing IGF1 protein level in GAD67^+^ neurons from the brain sections containing the PrL. **(D)** Quantification of IGF1 immunofluorescence intensity in GAD67^+^ and NeuN^+^ neurons (*n* = 4 mice/group, one-way ANOVA, *F*_(2, 9)_ = 53.76, *p* < 0.001). Data normalized to the young-adult group. Data are expressed as mean ± SEM. *, *p* < 0.05, **, *p* < 0.01, ***, *p* < 0.001. n.s., not significant.

IGF1 and IGF1R expressions in the brain decline with age; however, their cell-type-specific changes in the PrL remain underexplored. To address this, we examined IGF1 and IGF1R levels in the two major neuronal subtypes in the PrL, excitatory and inhibitory neurons, across young adult (2 months), middle-aged (9 months) and aged (15–18 months) mice. Using CaMKII (Liu and Jones, 1996; Sik et al., 1998) and GAD67 (Meyer et al., 2011) as markers for excitatory and inhibitory neurons, respectively, we tested the expression of IGF1 and IGF1R within these neuronal subtypes. We found that IGF1 expression was significantly reduced in both excitatory (Figures 1A,B) and inhibitory neurons (Figures 1C,D) from the PrL in middle-aged and aged C57BL/6 mice compared with young-adult controls. Interestingly, IGF1 levels in excitatory neurons are similar between middle-aged and aged mice (Figures 1A,B), whereas IGF1 levels in inhibitory neurons show further reduction in aged mice (Figures 1C,D).

**Figure 2:**
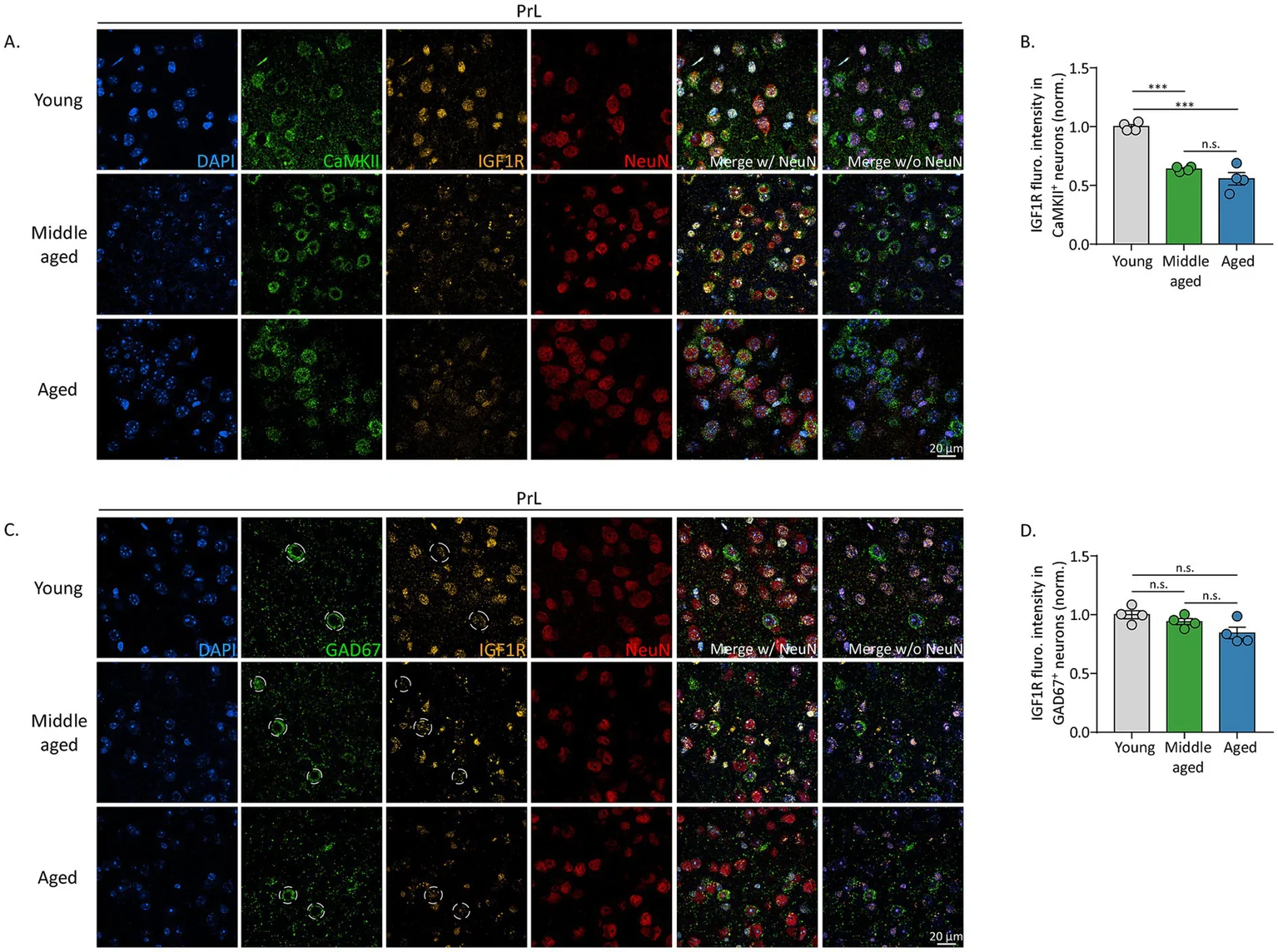
Aging induces cell-type-specific changes in IGF1R expression in the PrL **(A)** Representative immunohistochemical images showing IGF1R protein level in CaMKII^+^ and NeuN^+^ neurons from the brain sections containing the PrL. **(B)** Quantification of IGF1R immunofluorescence intensity in CaMKII^+^ neurons (*n* = 4 mice/group, one-way ANOVA, *F*_(2, 9)_ = 50.53, *p* < 0.0001). **(C)** Representative immunohistochemical images showing IGF1R protein level in GAD67^+^ and NeuN^+^ neurons from the brain sections containing the PrL. **(D)** Quantification of IGF1R immunofluorescence intensity in GAD67^+^ neurons (*n* = 4 mice/group, one-way ANOVA, *F*_(2, 9)_ = 4.22, *p* = 0.0509). Data normalized to young-adult group. Data are expressed as mean ± SEM. ***, *p* < 0.001. n.s., not significant.

In contrast, IGF1R expression showed a cell-type-dependent pattern (Figure 2). IGF1R expression was significantly dampened in excitatory neurons in both middle-aged and aged mice compared to young-adult mice, with a further reduction observed in aged mice compared to middle-aged mice (Figures 2A,B). Notably, IGF1R expression in inhibitory neurons remained unchanged among all age groups (Figures 2C,D). Collectively, these data suggest that IGF1 and IGF1R expressions in different cell types may be differentially altered during aging, especially in excitatory neurons.

### Reduced IGF1R in PrL excitatory neurons impairs anxiety-related, social, and cognitive behaviors in young adult mice

As we observed that aging is associated with cell-type-dependent reductions in IGF1 and IGF1R expressions in the PrL, we next asked whether disruption of IGF1-IGF1R interaction in young adult mice is sufficient to produce behavioral features associated with aging. We sought to explore whether inhibiting IGF1-IGF1R system during early adulthood alters behavior across multiple domains, including anxiety, sociability, and cognitive function. To achieve this, we utilized the *Igf1r* cKD mouse model as in our previous publication (Yue et al., 2022). By injecting AAV-CaMKII-Cre-mCherry virus into the PrL of *Igf1r^f/f^* mice, we induced excitatory neuron-specific knockdown of *Igf1r*, and the knockdown efficiency was validated by a significant reduction of IGF1R expression in mCherry^+^ neurons of *Igf1r* cKD mice compared to mice injected with control virus (Supplementary Figures 1A–C). Viral injections were conducted in 6-week-old mice, and behavioral assessments were performed 2 weeks later, corresponding to young adulthood in mice (Supplementary Figures 2A).

**Figure 3:**
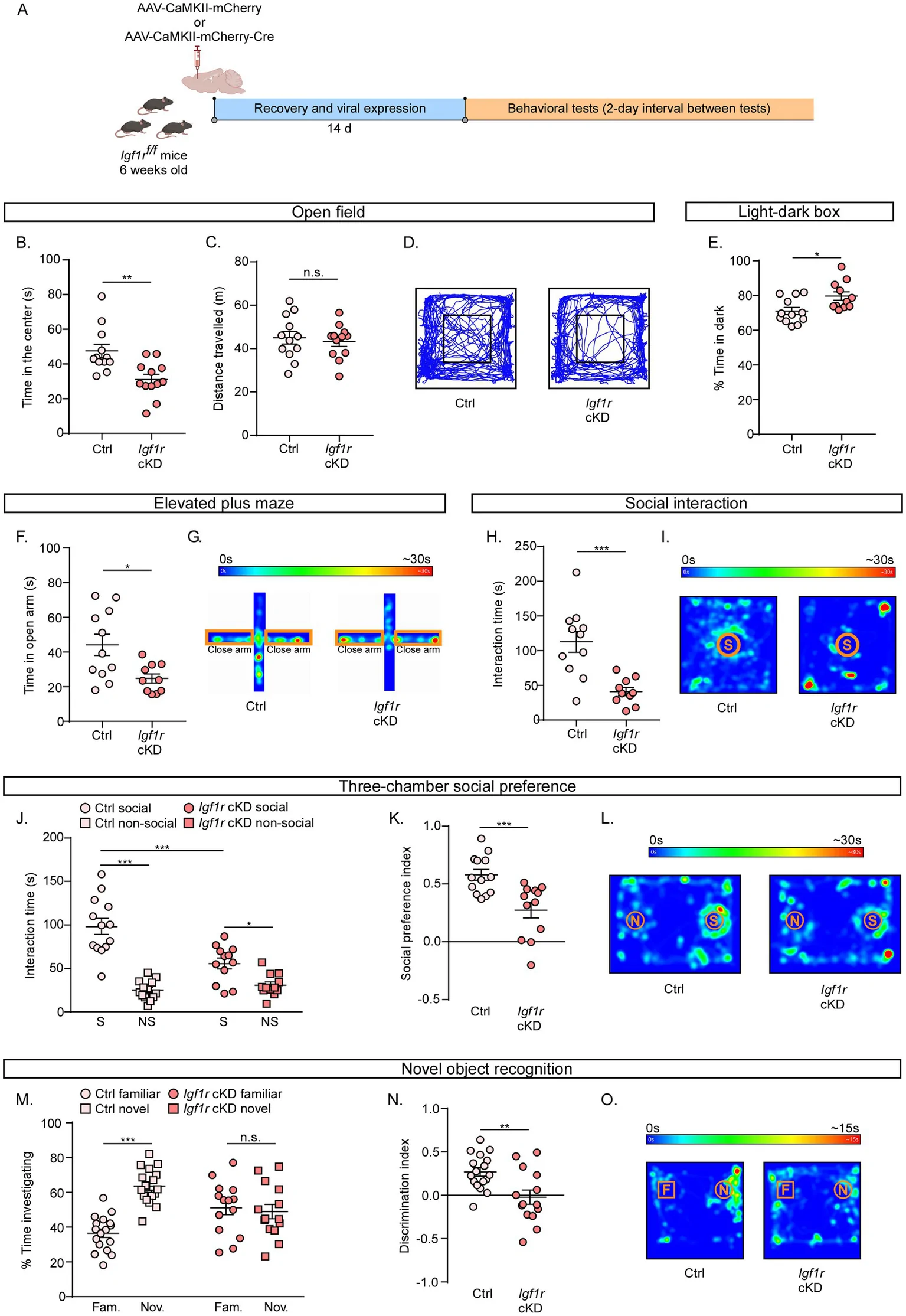
PrL excitatory neurons-specific IGF1R deficiency induces age-associated behavioral alterations. **(A)** Experimental time for viral injection, recovery and viral expression and behavioral tests in 6–8-week-old *Igf1r^f/f^* mice. **(B,C)** Averaged time spent in the center **(B)** and **(C)** distance traveled in open field test B: (*n* = 12 mice/group, unpaired *t*-test, *t*_22_ = 3.429, *p* = 0.0024. C: *n* = 12 mice/group, unpaired *t*-test, *t*_22_ = 0.4697, *p* = 0.6432. Ctrl: Mice injected with AAV-CaMKII-mCherry. *Igf1r* cKD: Mice injected with AAV-CaMKII-Cre-mCherry). **(D)** Representative tracks of open field exploration of Ctrl and *Igf1r* cKD mice. **(E)** Averaged percentage of time spent in the dark compartment in the light–dark box test (*n* = 11–12 mice/group, unpaired *t*-test, *t*_21_ = 2.721, *p* = 0.0128). **(F)** Averaged time spent in the open arm in elevated plus maze test (*n* = 10–11 mice/group, unpaired *t*-test, *t*_19_ = 2.816, *p* = 0.011). **(G)** Representative heat maps illustrating Ctrl and *Igf1r* cKD mice exploration in the elevated plus maze. **(H)** Averaged time spent interacting with the social stimulus (S) in the social interaction test (*n* = 10–11 mice/group, unpaired *t*-test, *t*_19_ = 4.242, *p* = 0.0004). **(I)** Representative heat maps depicting Ctrl and *Igf1r* cKD mice interacting with social stimulus in the social interaction test. **(J)** Averaged time spent interacting with the social stimulus (S) and non-social stimulus (NS) by Ctrl and *Igf1r* cKD mice in the three-chamber social preference test (*n* = 12–13 mice/group, two-way ANOVA, *F*_1, 23 (genotype)_ = 8.231, *p* = 0.0087, *F*_1, 23 (social stimulus)_ = 73.12, *p* < 0.0001). **(K)** Averaged social preference index of Ctrl and *Igf1r* cKD mice (*n* = 12–13 mice/group, unpaired *t*-test, *t*_23_ = 3.798, *p* = 0.0009). **(L)** Representative heat maps showing the exploration of Ctrl and *Igf1r* cKD mice in the three-chamber social preference test. **(M)** Averaged percentage of time spent investigating the familiar object (Fam.) and the novel object (Nov.) by Ctrl and *Igf1r* cKD mice in the novel object recognition test (*n* = 14–17 mice/group, two-way ANOVA, *F*_1, 29 (genotype)_ = 35.69, *p* < 0.0001, *F*_1, 29 (object)_ = 7.356, *p* = 0.0111). **(N)** Averaged discrimination index of Ctrl and *Igf1r* cKD mice (*n* = 14–17 mice/group, unpaired *t*-test, *t*_29_ = 3.186, *p* = 0.0034). **(O)** Representative heat maps illustrating the exploration of Ctrl and *Igf1r* cKD mice in the novel object recognition test (F: Familiar object. N: Novel object). Data are expressed as mean ± SEM. *, *p* < 0.05, **, *p* < 0.01, ***, *p* < 0.001. n.s., not significant.

We first examined anxiety-like behaviors in *Igf1r* cKD mice using the open field test, light–dark box test, and elevated plus maze test. As shown in Figure 3B, compared to mice injected with AAV-CaMKII-mCherry virus (Ctrl), *Igf1r* cKD mice demonstrated reduced time in the center of the open field with no significant changes in total distance traveled (Figures 3C,D). In the light–dark box test, *Igf1r* cKD mice spent more time in the dark compartment (Figure 3E) and showed a reduced number of transitions between compartments (Supplementary Figure 2B). In the elevated plus maze test, *Igf1r* cKD mice showed reduced time spent in the open arm (Figures 3F,G) and decreased number of open arm entries (Supplementary Figure 2C) compared to control animals. Taken together, these data suggest that reduced IGF1R expression in PrL excitatory neurons induces anxiety-like behavior during young adulthood, and this behavioral phenotype is less likely due to changes in overall exploration or locomotor activity, as there is no change in total distance traveled in the open field test.

Next, we explored the effect of *Igf1r* cKD on sociability. In the social interaction test, we found that *Igf1r* cKD mice spent significantly less time interacting with the social stimulus compared to control animals (Figures 3H,I). *Igf1r* cKD mice also showed significantly increased latency to interact with the social stimulus (Supplementary Figure 2D). In the test phase of the three-chamber social preference test, control mice spent significantly more time investigating the social stimulus than the non-social stimulus (Figure 3J), confirming that control mice displayed the expected preference for the social stimulus. *Igf1r* cKD mice also spent significantly more time investigating social stimulus, indicating that the social preference was retained. However, *Igf1r* cKD mice spent less total time investigating the social stimulus compared to control mice, suggesting attenuated social motivation rather than a complete loss of social preference. Correspondingly, the social preference index for *Igf1r* cKD mice was significantly decreased compared to control mice (Figure 3K). Together, these data suggest that reduced IGF1R levels in PrL excitatory neurons impair social behaviors in young adult mice.

To test the role of PrL IGF1R on cognitive function during early adulthood, we used the novel object recognition test. As shown in Figure 3M, control animals spent significantly more time investigating the novel object compared to the familiar object, indicating intact short-term recognition memory. In contrast, *Igf1r* cKD mice spent a similar amount of time investigating two objects and showed a reduced discrimination index compared to control animals (Figure 3N). Together, these data indicate that dampened IGF1R function in the PrL excitatory neurons impairs cognitive function in young adult mice.

### Local IGF1 treatment partially ameliorates behavioral impairments in aged mice through IGF1R in PrL excitatory neurons

**Figure 4:**
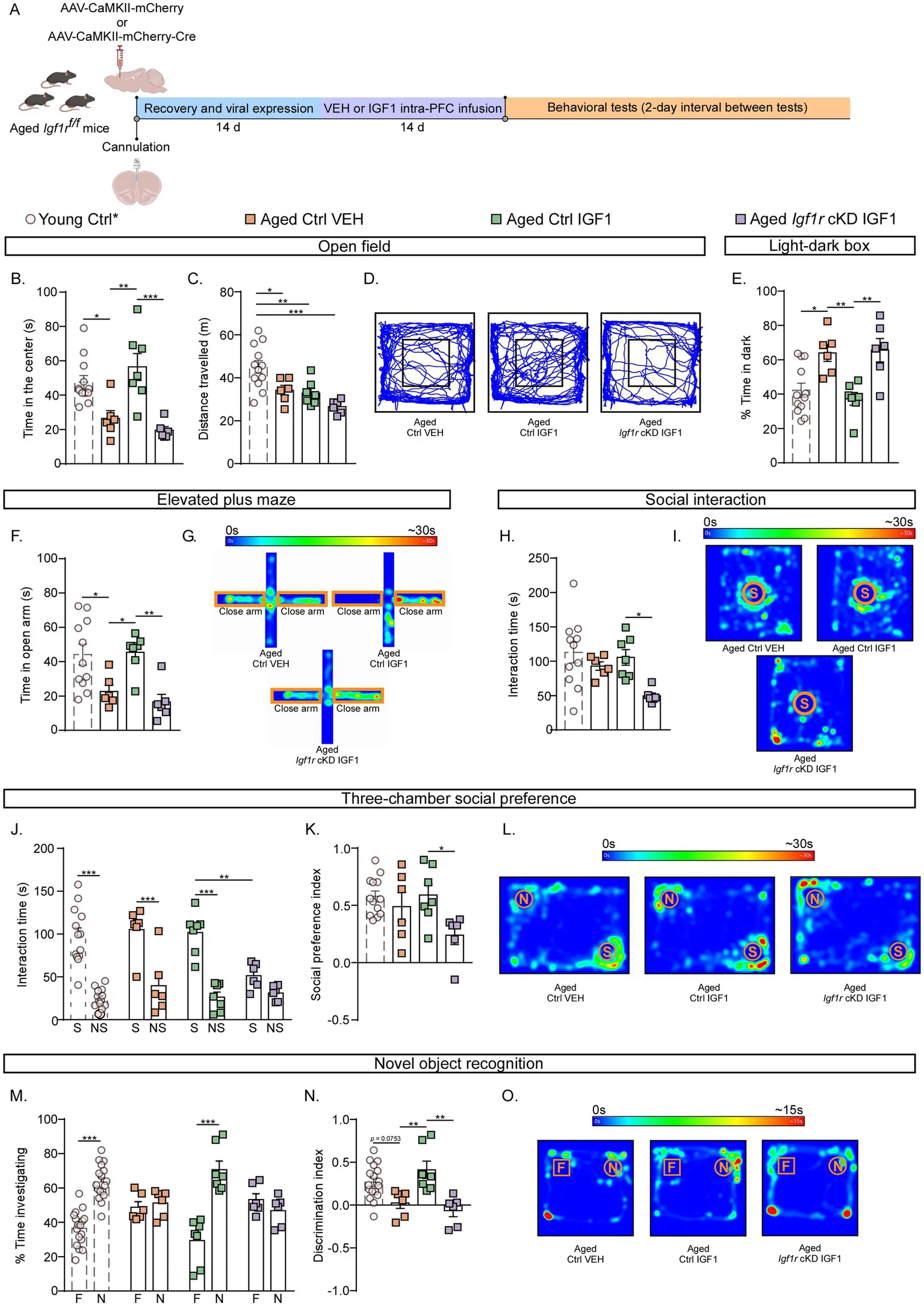
Intra-PrL IGF1 microinjection partially ameliorates age-related behavioral impairments through IGF1R signaling in excitatory neurons. **(A)** Experimental time for viral injection, cannulation, IGF1 treatment and behavioral tests in aged *Igf1r*^f/f^ mice. **(B)** Averaged time spent in the center and **(C)** distance traveled in open field test (**B**: *n* = 6–7 mice/group, one-way ANOVA, *F*_3, 27_ = 9.241, *p* = 0.002. **C**: *n* = 6–7 mice/group, one-way ANOVA, *F*_3, 27_ = 11.26, *p* < 0.0001. Young Ctrl*: the same dataset as in Figure 3, 6–8-week-old mice injected with AAV-CaMKII-mCherry. Aged Ctrl VEH: aged mice injected with AAV-CaMKII-mCherry and infused with saline. Aged Ctrl IGF1: aged mice injected with AAV-CaMKII-mCherry and infused with IGF1. Aged *Igf1r* cKD IGF1: aged mice injected with AAV-CaMKII-Cre-mCherry and infused with IGF1). **(D)** Representative tracks of open field exploration of Aged Ctrl VEH, Aged Ctrl IGF1 and Aged *Igf1r* cKD IGF1 mice. **(E)** Averaged percentage of time spent in the dark compartment in the light–dark box test (*n* = 6–7 mice/group, one-way ANOVA, *F*_3, 27_ = 8.121, *p* = 0.0005). **(F)** Averaged time spent in the open arm in elevated plus maze test (*n* = 6–7 mice/group, one-way ANOVA, *F*_3, 26_ = 6.903, *p* = 0.0014). **(G)** Representative heat maps illustrating Aged Ctrl VEH, Aged Ctrl IGF1 and Aged *Igf1r* cKD IGF1 mice exploration in the elevated plus maze. **(H)** Averaged time spent interacting with the social stimulus (S) in the social interaction test (*n* = 6–7 mice/group, one-way ANOVA, *F*_3, 26_ = 4.466, *p* = 0.0117). **(I)** Representative heat maps depicting Aged Ctrl VEH, Aged Ctrl IGF1 and Aged *Igf1r* cKD IGF1 mice interacting with social stimulus in the social interaction test. **(J)** Averaged time spent interacting with the social stimulus (S) and non-social stimulus (NS) in the three-chamber social preference test (*n* = 6–7 mice/group, two-way ANOVA, *F*_3, 28 (group)_ = 3.466, *p* = 0.0294, *F*_1, 28 (social stimulus)_ = 99.87, *p* < 0.0001). **(K)** Averaged social preference index (*n* = 6–7 mice/group, one-way ANOVA, *F*_3, 28_ = 3.759, *p* = 0.0219). **(L)** Representative heat maps showing the exploration of Aged Ctrl VEH, Aged Ctrl IGF1 and Aged *Igf1r* cKD IGF1 mice in the three-chamber social preference test. **(M)** Averaged percentage of time spent investigating the familiar object (F) and the novel object (N) in the novel object recognition test (*n* = 6–7 mice/group, two-way ANOVA, *F*_3, 32 (group)_ = 4.491, *p* = 0.0097, *F*_1, 32 (object)_ = 18.39, *p* = 0.0002). **(N)** Averaged discrimination index (*n* = 6–7 mice/group, one-way ANOVA, *F*_3, 32_ = 7.939, *p* = 0.0004). **(O)** Representative heat maps illustrating the exploration of Aged Ctrl VEH, Aged Ctrl IGF1 and Aged *Igf1r* cKD IGF1 mice in the novel object recognition test (F: familiar object. N: novel object). Data are expressed as mean ± SEM. *, *p* < 0.05, **, *p* < 0.01, ***, *p* < 0.001.

Next, we asked whether restoring IGF1 levels in the PrL could ameliorate age-related behavioral alterations and whether this effect depends on IGF1R in PrL excitatory neurons. To achieve this, aged control and *Igf1r* cKD mice received intra-PrL infusion of IGF1 (100 ng/μL, 1 μL/hemisphere) or vehicle (VEH) for 14 days (Figure 4A; Supplementary Figure 3A). To determine whether restoration of IGF1 levels ameliorates age-related anxiety-like behavior, we tested mice in the open field, elevated plus maze, and light–dark box tests. In the open field test, aged vehicle-treated control mice (Aged Ctrl VEH) spent significantly less time in the center than young control mice (Young Ctrl*, same dataset in Figure 3), indicating increased anxiety-like behavior during aging (Figure 4B). Chronic intra-PrL IGF1 infusion significantly increased center time in aged control mice (Aged Ctrl IGF1), indicating that restoration of IGF1 levels ameliorated the anxiety-like phenotype. Importantly, this recovery effect was significantly attenuated in aged mice with *Igf1r* cKD in the PrL excitatory neurons (Aged *Igf1r* cKD IGF1). This suggests that the action of IGF1 depends, at least in part, on IGF1R in excitatory neurons. Consistent with the open field results, aged vehicle-treated control mice showed increased anxiety-like behavior in both light–dark box (Figure 4E; Supplementary Figure 4A) and elevated plus maze tests (Figures 4F,G; Supplementary Figure 4B) compared with young controls. Chronic intra-PrL IGF1 infusion significantly alleviated these anxiety-like behaviors, whereas the restoration effects of IGF1 were significantly blunted in aged mice with *Igf1r* cKD in the PrL excitatory neurons. Notably, aged mice showed lower traveled distance in the open field test (Figures 4C,D), and IGF1 treatment improved anxiety-like behaviors without significantly changing locomotor activity, suggesting that the behavioral improvements did not result from changes in locomotion.

We then examined social behaviors using social interaction and three-chamber social preference tests. We found that aged vehicle-treated control mice did not show a significant reduction in sociability compared to young controls (Figures 4H–L; Supplementary Figure 4C). Consistently, intra-PrL IGF1 treatment did not alter social behaviors in aged control mice. However, aged *Igf1r* cKD mice receiving IGF1 showed reduced sociability compared to aged IGF1-treated control mice, which is consistent with our observations that *Igf1r* cKD impaired social behaviors in young mice (Figures 3H–L).

Finally, we evaluated cognitive function using the novel object recognition test. Aged vehicle-treated control mice exhibited impaired recognition memory compared to young controls (Figures 4M–O). Chronic intra-PrL IGF1 infusion restored recognition memory in aged control mice, whereas this effect was absent in aged *Igf1r* cKD mice, indicating that IGF1-mediated recovery of cognitive function relies on IGF1R in excitatory neurons.

## Discussion

The current study demonstrated cell-type-dependent IGF1 and IGF1R alterations in the aging PrL, marked by reduced IGF1 expression in both excitatory and inhibitory neurons and a selective decrease in IGF1R expression in excitatory neurons. This observation appears to be in discrepancy with prior studies reporting age-related increases in IGF1R in hippocampal and cortical regions, which have been interpreted as compensatory responses to declining IGF1 signaling (Ashpole et al., 2015; Chung et al., 2002; Moloney et al., 2010). Several reasons could contribute to these discrepancies. For example, some prior studies rely on bulk tissue analyses, which lack brain-region resolution. Some studies focus on hippocampus and cerebral cortex, regions showing distinct molecular aging profiles from the PrL. The hippocampus, a region characterized by ongoing adult neurogenesis detectable in aged rodents (Spalding et al., 2013; Kuhn et al., 1996), may engage compensatory upregulation of IGF1R to support plasticity. In contrast, the PrL, where neurogenesis is extremely low in adulthood (Ming and Song, 2011), may adopt a different regulatory strategy. Compared to other subcortical regions, the PrL is among the last to mature (Casey et al., 2005), with its development extending into adolescence and early adulthood (Gogtay et al., 2004). IGF1 signaling plays a critical role in these neurodevelopmental processes (Dyer et al., 2016; Carson et al., 1993). The prolonged reliance of the PrL on IGF1/IGF1R signaling during development may render it particularly vulnerable to age-related declines in IGF1 signaling, potentially limiting its capacity for compensatory upregulation compared to neurons in earlier-maturing brain regions. In addition, temporal dynamics may also contribute to these differences. Our study examined C57BL/6 mice across an age range of 2 to 18 months, with 18-month-old mice representing an early stage of aging in the mouse lifespan (Shoji et al., 2016; Yanai and Endo, 2021). In contrast, compensatory upregulation of brain IGF1R reported in prior studies has primarily been observed at more advanced ages (e.g., ≥24 months). These findings highlight that the CNS IGF1/IGF1R signaling may show brain region and/or cell-type-specific dynamic regulation during aging. Future studies are needed to further define the spatial and longitudinal dynamics of brain IGF1/IGF1R signaling and its functional role in brain aging.

IGF1 has been shown to enhance excitability and synaptic plasticity of PFC pyramidal neurons, processes involved in normal prefrontal circuit function and cognitive and affective behaviors (Maglio et al., 2021). Therefore, changes in IGF1 and IGF1R expressions in PrL excitatory neurons during aging may compromise their function and contribute to age-associated behavioral impairments. Our finding demonstrates that adult-onset PrL *Igf1r* cKD leads to certain age-associated behavioral features, including increased anxiety-like behavior and cognitive impairment (Figure 3), and that local IGF1 infusion in aged mice attenuates these impairments (Figure 4). These observations are consistent with previous studies associating diminished IGF1-IGF1R system with cognitive and affective dysfunction. Circulating IGF1 deficiency resulting from liver-specific *Igf1* knockdown during young adulthood in mice impairs spatial learning by disrupting neurovascular coupling and reducing activity-dependent cerebral blood flow (Toth et al., 2015). Intranasal IGF1 treatment in aged mice has been shown to improve spatial and working memory and reduce anxiety- and depressive-like behaviors (Farias Quipildor et al., 2019), supporting the role of circulating IGF1 in age-associated behavioral impairments. In addition, local production of IGF1 is also critical. For example, brain-wide conditional deletion of neural *Igf1* abolishes hippocampal long-term potentiation and attenuates spatial memory despite normal circulating IGF1 levels, indicating that locally synthesized IGF1 may function independently of the systemic pool. These dysfunctions are accompanied by an altered excitatory/inhibitory balance, suggesting that local IGF1 is critical for maintaining normal circuit function (Herrero-Labrador et al., 2023).

Despite these important findings, several key questions remain unresolved. Previous studies lack the brain-region and cell-type specificity needed to identify the sites of IGF1 action. Moreover, systemic IGF1 deficiency models cannot determine whether the observed behavioral alterations result from direct neuronal IGF1 signaling or from indirect metabolic and cerebrovascular alterations. Similarly, brain-wide neural *Igf1* deletion disrupts IGF1 signaling from early development, making it difficult to distinguish the role of IGF1 during brain development from its role in maintaining neuronal function in adulthood. In addition, previous studies have largely focused on hippocampal neurons and cognitive function, with less attention to anxiety-like behavior or sociability. Our study addresses these gaps by selectively manipulating IGF1R in PrL excitatory neurons during adulthood, thereby avoiding developmental confounds while providing both brain-region and cell-type specificity. We demonstrate that downregulation of IGF1R in this neuronal population is sufficient to induce anxiety-like behavior and cognitive impairment, and that local IGF1 infusion ameliorates these age-related behavioral alterations in an IGF1R-dependent manner, except for social investigation.

Age-related changes in social behavior are more variable. Previous rodent studies have reported heterogeneous age-related changes in social behaviors, with some functions declining (Shoji and Miyakawa, 2019; Gerasimenko et al., 2020) while others remain stable or even improve (Greene et al., 2023; Yamada et al., 2025). This divergence may depend on mouse strain, specific age and behavioral paradigm examined. Similarly, human studies also indicate that aging is associated with multidirectional changes in several social cognition domains (Grainger et al., 2023). Together, these findings suggest that social behavior is not uniformly affected by aging but instead exhibits domain-specific and context-dependent alterations. Accordingly, the social interaction and three-chamber social preference tests used in the present study, which primarily assess sociability and social approach, may not capture all aspects of age-related social dysfunction. This may explain why aged mice did not exhibit detectable social impairment under the current experimental conditions. In contrast, reduced social behavior is observed only in the aged *Igf1r* cKD group, consistent with the phenotype of young *Igf1r* cKD mice (Figures 3H–L), suggesting that IGF1-IGF1R system may be more sensitive than aging at revealing changes in social behavior within the current social interaction and three-chamber social preference paradigms. Indeed, IGF1/IGF1R signaling has been implicated in neurodevelopmental disorders, in which social deficits are a hallmark phenotype (Pini et al., 2012; Deacon et al., 2015; Castro et al., 2014; Provenzano et al., 2014). Thus, the effects of *Igf1r* cKD on social behavior may reflect a broader role of IGF1R in regulating social function rather than an aging-specific mechanism. However, this broader role does not exclude the possible contribution of IGF1/IGF1R to aging. Future studies incorporating more comprehensive behavioral paradigms, such as social recognition and social novelty, together with additional aging models, will be important for defining the full spectrum of age-associated social changes and determining how the IGF1/IGF1R system contributes to these processes. Despite these limitations, our data consistently demonstrate that IGF1R in PrL excitatory neurons is required to maintain normal social behavior in both young and aged mice. Together with its role in anxiety-like behavior and cognition, these findings identify IGF1R in PrL excitatory neurons as a key mediator of selected aspects of behavioral dysfunction during aging.

Another limitation of the current study is that cognitive function was assessed using the novel object recognition (NOR), which primarily measures familiarity-based recognition memory and relies on the PFC for encoding contextual discrimination (Morici et al., 2015; Farovik et al., 2008). However, cognitive aging encompasses a substantially broader range of functions that a single recognition memory task cannot fully capture. For example, spatial memory and temporal order memory additionally engage hippocampal circuits, with contributions from the PFC (Morrison and Baxter, 2012). Beyond memory, PFC-dependent executive functions, including attention, cognitive flexibility, and set-shifting, are also vulnerable to aging (Zanto and Gazzaley, 2019; Raz et al., 2005). The impact of PFC IGF1 and IGF1R on these dimensions of cognitive function requires further investigation. Additionally, our study focused on broad excitatory and inhibitory neuronal populations and does not assess the diversity of interneuron subtypes or non-neuronal cells. It’s been suggested that microglia retain the capacity to produce IGF1 in aged AD mouse model (Myhre et al., 2019), and IGF1 exert anti-inflammatory by inhibiting the astrocytic response to inflammatory stimuli and promoting the microglial M2 phenotype (Labandeira-Garcia et al., 2017). This reveals the possibility that IGF1-IGF1R system is also differentially regulated in glial cells in the aging PFC. In addition, IGF1 not only acts on IGF1R but also stimulates insulin receptors (IRs) (Belfiore et al., 2017; LeRoith and Roberts, 2003), and IRs are also involved in synaptic plasticity (Ferrario and Reagan, 2018; Fetterly et al., 2021). More studies are needed to elucidate how the IGF1 system interacts across different cell types via distinct receptors during aging. Furthermore, although our study included both sexes, it did not systematically evaluate sex differences. Previous studies have reported sex-dimorphic expression patterns of IGF1 and IGF1R and their sex-specific role in brain aging (Farias Quipildor et al., 2019; Waters et al., 2003; Holzenberger et al., 2003). Therefore, future studies are needed to determine whether these findings are sex dependent.

In conclusion, our brief report demonstrates a previously unrecognized, cell-type-dependent IGF1 and IGF1R disruption in the aging PrL, defined by a shared reduction in IGF1 across excitatory and inhibitory neurons and a selective loss of IGF1R in excitatory neurons. Functional manipulation further indicates that selective reduction of IGF1R in PrL excitatory neurons is sufficient to induce behavioral alterations across cognitive, social, and anxiety-related domains. Moreover, enhancement of PrL IGF1 level attenuates age-associated behavioral impairments through IGF1R in excitatory neurons. Together, these findings highlight a critical role for cell-type-specific IGF1-IGF1R system in brain aging and suggest targeting this pathway as a strategy for preserving PFC function.

## Data Availability

The original contributions presented in the study are included in the article/Supplementary material, further inquiries can be directed to the corresponding author.
