# Supplementary material for: Cell-type-dependent decline of IGF1 and IGF1R in the prefrontal cortex contributes to age-related behavioral changes
Source: Front Aging Neurosci. 2026 Aug 19;18:1858227. doi: 10.3389/fnagi.2026.1858227 (PMC13533932; doi:10.3389/fnagi.2026.1858227)
Supplement: Supplementary file 1 [file Data_Sheet_1.docx]

**Supplementary material**

**Cell-type-dependent decline of IGF1 and IGF1R in the prefrontal cortex contributes to age-related behavioral changes**

Shuwen Yue^1^, Christian Young^1^, Arushi Garg^1^, Tyler Sarovich^1^, Lu Chen^1^, Kendall Dickerson^1^, Zi-Jun Wang^1,2^

1Department of Pharmacology & Toxicology, School of Pharmacy, University of Kansas, Lawrence, KS, USA

2Cofrin Logan Center for Addiction Research and Treatment, University of Kansas, Lawrence, KS, USA

*Corresponding author. Department of Pharmacology and Toxicology, School of Pharmacy, University of Kansas, 1251 Wescoe Hall Drive, Lawrence, KS 66045, USA. E-mail address: zjwang@ku.edu (Z.-J. Wang).

**Supplementary Methods**

**Animal**

C57BL/6J mice (stock number 000664), *Igf1r*^flox/flox^ (*Igf1r*^f/f^) mice (B6;129-*Igf1r^tm2Arge^*/J, 012251) and Vgat-ires-cre (*Vgat*-Cre) mice (B6J.129S6(FVB)-*Slc32a1^tm2(cre)Lowl^*/MwarJ, 028862) were purchased from Jackson lab. Both male and female mice were used in this study. All animals were maintained on a 12-hour light/dark cycle (lights off at 11:00 A.M. and light on at 11:00 P.M.) with ad libitum access to water and food chow. All animal experiments were performed under the approval of the Institutional Animal Care and Use Committee, University of Kansas. All animals were maintained according to the National Institutes of Health guidelines in Association for Assessment and Accreditation of Laboratory Animal Care accredited facilities.

**Immunohistochemistry** **and quantifications**

Three age groups were used: young-adult (2 months), middle-aged (9 months) and aged mice (15-18 months). Mice were transcardially perfused with 0.01 M PBS, pH 7.4 (phosphate-buffered saline) followed by 4% paraformaldehyde (PFA) in PBS. Brains were collected and post-fixed in 4% PFA at 4 °C for 24 h, paraffin embedded and sectioned coronally at 7 µm through the PrL. For immunostaining, sections were incubated with primary antibody IGF1 (1:100, Novus Biologicals, NBP2-16929), IGF1R (1:100, Cell Signaling Technology, #3027), GAD67 (1:50, Millipore, MAB5406), CaMK2A (1:50, Santa Cruz, sc-13141) or NeuN (1:100, Millipore, ABN91) overnight at 4 °C. On the second day, sections were incubated with anti-Alexa Fluor 488-conjugated, anti-Alexa Fluor 555-conjugated or anti- Alexa Fluor 647-conjugated secondary antibody at 1:200 (Thermo Fisher) for 2 h at room temperature. Images were taken on a Nikon Ti2 Eclipse microscope. IGF1 and IGF1R immunofluorescence intensities in the CaMKII^+^ and GAD67^+^ neurons within the PrL were quantified in ImageJ by identifying cells positive for the respective markers, as previously reported (1, 2). Immunofluorescence signals were averaged across four images per animal to obtain a single value for each animal (n = 4 animals/group), which was used for statistical analysis.

**Stereotaxis surgery and IGF1 microinjection**

To induce *Igf1r* conditional knockdown, *Igf1r^f/f^* mice (carrying *loxP* sites flanking exon 3 of the *Igf1r* gene (3)) were deeply anesthetized with 100 mg/kg ketamine as well as 5 mg/kg xylazine and placed on a stereotaxic apparatus (RWD instruments). rAAV-CaMKIIa-mCherry-P2A-CRE-WPRE-hGH polyA (titer: 2X10^12^ VG/mL, #PT-0739, BrainVTA) or control virus rAAV-CaMKIIa-mCherry-WPRE-hGH polyA (titer: 2X10^12^ VG/mL, #PT-0108, BrainVTA) was delivered bilaterally (300 nL/side) into the PrL (AP: +2.0, ML: ±0.3, DV: -2.0). The injection was controlled by a minipump (KD Scientific) at the rate of 50 nL/min. The injection needle (10 uL, Hamilton) was kept in place for another 10 minutes for full dispersion of the virus. Behavioral experiments or tissue collection were performed after 14 days of viral expression.

For IGF1 infusion experiments, cannulation was performed at the same time as viral injection. Based on previous reports (4, 5), a double guide cannula with a dummy (RWD Instruments) was implanted into the PrL (AP: +2.0, ML: ±0.3, DV: -1.5) and fixed with dental cement. A microinjector with a 0.5-mm protrusion below the guide cannula aiming at PrL (−2.0 mm) was used for infusion (**sFig. 3A**). Mice were assigned to receive saline (VEH) or IGF1 (100 ng/μL) infusion (1 uL/hemisphere, 0.5 µL/min using 100-μL Hamilton syringes) controlled by a syringe pump (RWD Instruments). After infusion, the needle was kept at the injection site for another 5 mins to allow complete diffusion.

**Behavioral test**

8-week-old control and *Igf1r* cKD animals were subjected to a series of behavioral tests in the order shown in **sFig. 2A**. All behavioral assessments were conducted by investigators blinded to group assignment and performed during the light phase. Animals were habituated to the testing room for at least 30 minutes prior to the test. Apparatuses were cleaned with disinfectants between each trial. A 2-days interval was maintained between consecutive behavioral tests. All tests were recorded and analyzed using ANY-maze tracking system and subsequently reviewed by investigators blinded to group allocation.

***Open field***

Mice locomotor and anxiety-like behaviors were tested in an open field transparent plastic apparatus (50×50×35 cm) for 15 minutes. At the beginning of the test, a mouse was gently placed in a corner square with its head facing the corner. Distance traveled and time in the center (20×20 cm) within 15 minutes were recorded and analyzed.

***Light-dark box***

The light-dark apparatus used in the current study contained a lit compartment (35×35×30 cm) and a dark compartment (35×35×30 cm) joined by a 7×12 cm doorway. The lit compartment was illuminated at 200 lux using an overhead LED light. Mouse was gently placed in the center of the lit compartment and allowed to move freely between two compartments for 10 minutes. Time spent in the dark chamber and number of transitions were recorded.

***Elevated plus maze***

The elevated plus maze used in the current study was a plus-shaped apparatus with four arms (7×40 cm) raised 50 cm above the ground. Two arms were enclosed with 30 cm tall opaque walls (closed arm) while the two open arms did not have walls. The test mouse was placed at the junction of the four arms of the maze facing the open arm and allowed to explore the maze for 5 minutes. Time spent in the open arm and open arm entries were recorded.

***Social interaction***

As previously described (6), the test mouse was habituated in an apparatus (50×50×35 cm) with a capsule (an inverted pencil cup) placed in the center for 10 minutes to prevent novelty-induced anxiety. After habituation, the mouse was returned to its home cage. The apparatus was then cleaned with disinfectant, and an age-, sex- and strain-matched mouse was placed inside the capsule as a social stimulus. The test animal was reintroduced into the apparatus to explore for 10 minutes. The latency to interact and time spent by the test animal interacting with the social stimulus, such as sniffing or being in close proximity to the capsule, was recorded and analyzed.

***Three-chamber social preference***

The three-chamber social preference test was performed as previously described with some modifications (7). Briefly, the test mouse was first habituated in a Plexiglass arena (75×50×35 cm) containing two capsules for 10 minutes. After habituation, the mouse was returned to its home cage for 5 minutes. The mouse was then reintroduced to the apparatus for a 10-min trial in which the capsules contained two identical objects. The animal was then returned to its home cage for 5 minutes. For the social preference test phase, the animal was placed into the apparatus for a 10-min trial, in which one capsule contained a novel object (non-social stimulus) while the other contained an age-, sex- and strain-matched mouse (social stimulus). The amount of time spent on the test animal interacting with each stimulus was recorded. The preference index was calculated as: (social time − non-social time) / (social time + non-social time).

***Novel object recognition***

The test mouse was placed in the apparatus (50×50×35 cm) for 5-min habituation, then returned to its home cage. The mouse was then placed back into the apparatus with two identical objects for 5 min, followed by 1-h retention interval in the home cage. For the test phase, the mouse was allowed to explore the apparatus containing one of the original objects (familiar) and a new object (novel) for 5 min, during which the percentage of time spent interacting with each object was scored. Discrimination ratio was calculated as: (novel-object time − familiar-object time) / (novel-object time + familiar-object time).

**Statistical analysis**

All statistical analyses were performed with GraphPad Prism 9.0 and Statistical Package for the Social Sciences (SPSS). Data were analyzed using one-way or two-way ANOVA as appropriate, followed by Bonferroni post hoc tests for multiple comparisons. Experiments with two groups were analyzed using two-tailed unpaired *t*-tests. All data are presented as the mean ± SEM, with *p* < 0.05 indicating significance. The number of animals in each group was provided in figure legends. Statistical details are provided in **Supplementary Table 1**.

**Supplementary Figure 1. PrL CaMKII^+^ neurons-specific *Igf1r* knockdown validation.** (**A**) Schematic showing *Igf1r^f/f^* mice injected with AAV-CaMKII-Cre-mCherry virus into the PrL to induce excitatory neuron-specific *Igf1r* conditional knockdown (cKD). (**B**) Representative immunohistochemical images showing IGF1R protein level in CaMKII^+^ neurons from the brain sections containing the PrL (Ctrl: mice injected with AAV-CaMKII-mCherry. *Igf1r* cKD: mice injected with AAV-CaMKII-Cre-mCherry). (**C**) Quantification of IGF1R immunofluorescence intensity in CaMKII^+^ neurons (n = 3 mice/group, unpaired *t*-test, *t*_4_ = 10.88, *p* < 0.0001).

**Supplementary Figure 2.** **PrL excitatory neurons-specific IGF1R deficiency induces aging-associated behavioral alterations. (A)** Experimental time for viral injection, recovery and viral expression and sequence of behavioral tests in 6-8-week-old *Igf1r^f/f^* mice. (**B**) Averaged transition numbers in the light-dark box test (n = 11-12 mice/group, unpaired *t*-test, *t*_21_ = 2.836, *p* = 0.0099). (**C**) Averaged number of open arm entries in the elevated plus maze tests (n = 10-11 mice/group, unpaired *t*-test, *t*_19_ = 3.41, *p* = 0.0029). (**D**) Averaged latency to interact with the social stimulus in the social interaction test (n = 10-11 mice/group, unpaired *t*-test, *t*_19_ = 3.103, *p* = 0.0059).

**Supplementary Figure 3. Validation for cannula location.** (**A**) Coronal brain section showing track of guide cannula aiming at the PrL. Verification experiments were conducted in n = 3 mice demonstrating similar results. Scale bar, 200 µm.

**Supplementary Figure 4. Intra-PrL IGF1 treatment ameliorates aging-related behavioral impairments through IGF1R signaling in pyramidal neurons.** (**A**) Averaged transition numbers in the light-dark box test (n = 6-7 mice/group, one-way ANOVA, *F*_3, 27_ = 5.914, *p* = 0.0031. Young Ctrl*: the same dataset presented in Figure 3, 6-8-week-old mice injected with AAV-CaMKII-mCherry. Aged Ctrl VEH: aged mice injected with AAV-CaMKII-mCherry and infused with saline. Aged Ctrl IGF1: aged mice injected with AAV-CaMKII-mCherry and infused with IGF1. Aged *Igf1r* cKD IGF1: aged mice injected with AAV-CaMKII-Cre-mCherry and infused with IGF1). (**B**) Averaged number of open arm entries in the elevated plus maze tests (n = 6-7 mice/group, one-way ANOVA, *F*_3, 26_ = 4.388, *p* = 0.0126). (**C**) Averaged latency to interact with the social stimulus in the social interaction test (n = 6-7 mice/group, one-way ANOVA, *F*_3, 26_ = 2.463, *p* = 0.0849).

Reference

1.	Yue S, Wang Y, Wang ZJ. Insulin-like growth factor 1 regulates excitatory synaptic transmission in pyramidal neurons from adult prefrontal cortex. Neuropharmacology. 2022;217:109204.

2.	Wang Y, Yue S, Yang F, Chen L, Singh A, Marciniak M, et al. Prelimbic cortex to ventral tegmental area projection regulates early social isolation stress-potentiated heroin seeking in mice. Nat Commun. 2025;16(1):9541.

3.	Dietrich P, Dragatsis I, Xuan S, Zeitlin S, Efstratiadis A. Conditional mutagenesis in mice with heat shock promoter-driven cre transgenes. Mamm Genome. 2000;11(3):196-205.

4.	Li G, Yue S, Wang Y, Singh A, Wang ZJ. IGF-1 Microinjection in the Prefrontal Cortex Attenuates Fentanyl-Seeking Behavior in Mice. Int J Neuropsychopharmacol. 2023;26(5):359-71.

5.	Wang ZJ, Shwani T, Liu J, Zhong P, Yang F, Schatz K, et al. Molecular and cellular mechanisms for differential effects of chronic social isolation stress in males and females. Mol Psychiatry. 2022;27(7):3056-68.

6.	Wang Y, Liu J, Yue S, Chen L, Singh A, Yu T, et al. Prefrontal cortex excitatory neurons show distinct response to heroin-associated cue and social stimulus after prolonged heroin abstinence in mice. Neuropsychopharmacology. 2025;50(8):1284-97.

7.	Rein B, Ma K, Yan Z. A standardized social preference protocol for measuring social deficits in mouse models of autism. Nat Protoc. 2020;15(10):3464-77.
